# Application of Python-Based Abaqus Secondary Development in Laser Shock Forming of Aluminum Alloy 6082-T6

**DOI:** 10.3390/mi15040439

**Published:** 2024-03-25

**Authors:** Junru Yang, Tongle Zhang, Chuijiang Kong, Boyu Sun, Ran Zhu

**Affiliations:** 1 College of Mechanical and Electronic Engineering, Shandong University of Science and Technology, Qingdao 266590, China; jryangzhang@163.com (J.Y.); ztl19981125@163.com (T.Z.); kongchuijiang@163.com (C.K.); 2 Shenyang Institute of Automation, Chinese Academy of Sciences, Shenyang 110016, China; sunboyu@sia.cn; 3 Key Laboratory of Urban Rail Transit Intelligent Operation and Maintenance Technology & Equipment of Zhejiang Province, Jinhua 321004, China

**Keywords:** Abaqus secondary development, python, laser shock forming, aluminum alloy 6082-T6

## Abstract

Aluminum alloy 6082-T6 is an important material for manufacturing the outer skin of high-speed trains, and laser shock forming can realize the rapid forming of complex-shaped plates. In order to improve the efficiency of the simulation modeling of laser shock forming for aluminum alloy 6082-T6, Python scripting language was used for the secondary development of Abaqus. A plugin was utilized to simulate and analyze the laser shock forming process of aluminum alloy 6082-T6. The coordinates of the plate after laser impact molding were measured using a coordinate measuring machine to calculate the arc bow height of the plate. The accuracy of the simulation model was verified by comparing with the simulation results. The deformation characteristics of plastic strain and arc height of aluminum alloy 6082-T6 under different laser process parameters were analyzed. The simulation plugin has a concise interface, high operability, and accurate results with the other parameters unchanged. When the laser energy is 5 J, 6 J, and 7 J, the corresponding arc heights are 5.9 mm, 6.6 mm, and 7.2 mm, respectively. As the thickness of the sheet increases, the deformation changes from concave at 1 mm to convex at 2 mm, 3 mm, 4 mm, and 5 mm. As the spot size increases from 1 mm to 5 mm, the transmission mode of the shock wave gradually changes from spherical wave to planar wave, and the arc height of the sheet increases from 4.6 mm to 8.2 mm. With the increase in the spot overlap rate, the impact area accumulates residual stress, and the arc height of the sheet is 5.7 mm, 6.6 mm, 7.3 mm, and 8.5 mm, respectively. The secondary development of ABAQUS 2021 using Python 3.6 scripting language has improved the efficiency of simulation modeling and provided reference for rapidly predicting the deformation characteristics of aluminum alloy 6082-T6 under different laser process parameters.

## 1. Introduction

**Figure 1:**
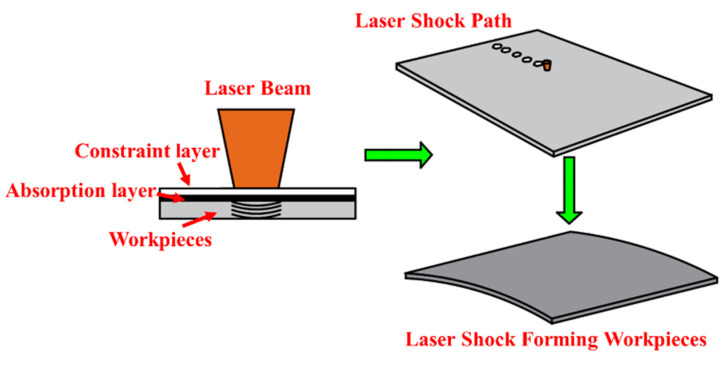
Schematic diagram of laser shock forming principle.

Aluminum alloy 6082-T6 is an important material for manufacturing high-speed train front-end skins. Due to high mold costs, the current method for manufacturing small batch train front-end skins mainly relies on manual and machine-assisted methods, which have problems such as low efficiency, insufficient accuracy, and poor performance of formed sheets. Laser shock forming is a new technology for moldless forming. It uses laser-induced plasma to generate shock waves. When the shock wave pressure exceeds the material’s dynamic yield strength, the material undergoes plastic deformation, ultimately achieving sheet bending deformation. This method can achieve rapid forming of large-sized and complex-shaped parts, and has the characteristics of high forming limits and large forming flexibility [1,2,3], as shown in Figure 1. At the same time, intense plastic deformation occurs in the impact zone, forming a certain depth of the residual compressive stress layer, which significantly improves the material’s wear resistance and fatigue resistance [4,5]. Previous researchers have conducted a series of experiments and simulation studies on laser shock forming. Rao et al. [6] found that residual compressive stress induced by lasers on the metal material surface is the driving force for sheet bending deformation. Increasing the impact area and overlap rate of the beam spot increases the deformation of the sample without changing the deformation mode. Yu et al. [7] studied the deformation of aluminum alloy 2024 under rectangular beam conditions and established a mathematical model for target bending deformation based on a double-coordinate equation. Morales et al. [8] found that if the applied pulse number has a stable quasi-proportional relationship, the bending deformation of the sample can be controlled within a certain range. Hu et al. [9] used the inherent strain obtained from the simulation of laser shock forming as the initial strain to elastically analyze residual stress and deformation field. Luo [10], based on the theory of inherent strain, derived the moment equation of bending deformation by partial differential equation and proposed a new physical quantity–inherent moment to describe the small curvature bending deformation of the sheet after laser shock forming, providing a new predictive model for complex deformation. Gachegova et al. [11] studied the influence of laser shock treatment on the fatigue life of titanium alloys. The study showed that after laser shock treatment, the fatigue fracture mechanism of the sample changed during low-cycle fatigue and high-cycle fatigue processes, significantly improving the fatigue life of the sample. Chen Fei et al. [12] used the Python scripting language to perform secondary development on ABAQUS and studied the mechanical shot peening treatment of aerospace components, improving the efficiency of simulation analysis. Rong Guangxu et al. [13] used the file processing method in Python to modify keywords and control the range of variable changes. Li et al. [14] used Python scripting to perform secondary development on ABAQUS and automatically modeled the microstructure of composite materials using code. Zhang et al. [15] used Python scripting to perform secondary development on ABAQUS and developed a plugin program for three-dimensional bending simulation analysis, which can construct geometric models.

Currently, there are few reports on the research of numerical simulation for laser shock forming based on the secondary development of the Abaqus preprocessing stage using Python scripting language. In this study, based on the theory of inherent strain, aluminum alloy 6082-T6 was taken as the research object. Python scripting language was used for the secondary development of Abaqus to parametrically establish a simulation model for laser shock forming of aluminum alloy 6082-T6. The forming law of aluminum alloy 6082-T6 under different process parameter treatments was investigated.

## 2. Finite Element Model

### 2.1. Inherent Strain Theory

Intrinsic strain refers to the strain that remains within a material or object after undergoing thermal cycling or stress loading [16,17]. Intrinsic strain is the cause of residual stress and deformation. It is equal to the total strain of an object minus the elastic strain and consists of thermal strain, volumetric strain, and plastic strain [18], as shown in Equation (1):

(1) $$\varepsilon^{*}=\varepsilon_{z}-\varepsilon_{e}=\varepsilon_{P}+\varepsilon_{T}+\varepsilon_{X}$$

In the above expression, ε* represents intrinsic strain, *ε*_z_ represents total strain, *ε*_e_ represents elastic strain, *ε*_P_ represents plastic strain, *ε*_T_ represents thermal strain, and *ε*_X_ represents phase strain.

In the laser shock forming process, thermal effects are not considered, so intrinsic strain can be regarded as the sum of plastic strain and residual volumetric strain. For some materials, such as low carbon steel and aluminum alloys, if phase transformation strain is not considered, intrinsic strain is equal to plastic strain.

(2) $$\varepsilon^{*}=\varepsilon_{P}$$

**Figure 2:**
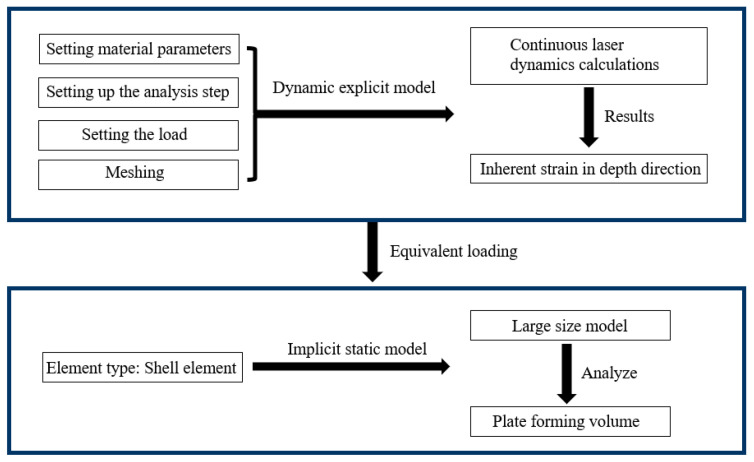
Modeling process of laser shock forming based on inherent strain.

The modeling process of laser shock forming is shown in Figure 2. Firstly, a dynamic display model is established using Python to set the material parameters, analysis time steps, loads, and grids. Then, a dynamic analysis using a small number of spots is performed to calculate the intrinsic strain in the depth direction. Finally, the obtained intrinsic strain is applied to the implicit static model using Python, and the forming amount of the plate after laser shock is predicted through static analysis.

### 2.2. Shock Wave Pressure and Material Constitutive Model

#### 2.2.1. Shock Wave Pressure Model

**Figure 3:**
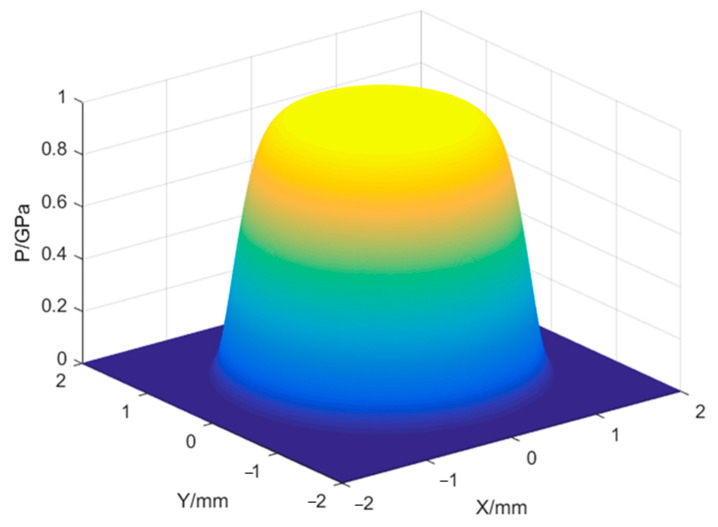
Space distribution of shock wave pressure.

**Figure 4:**
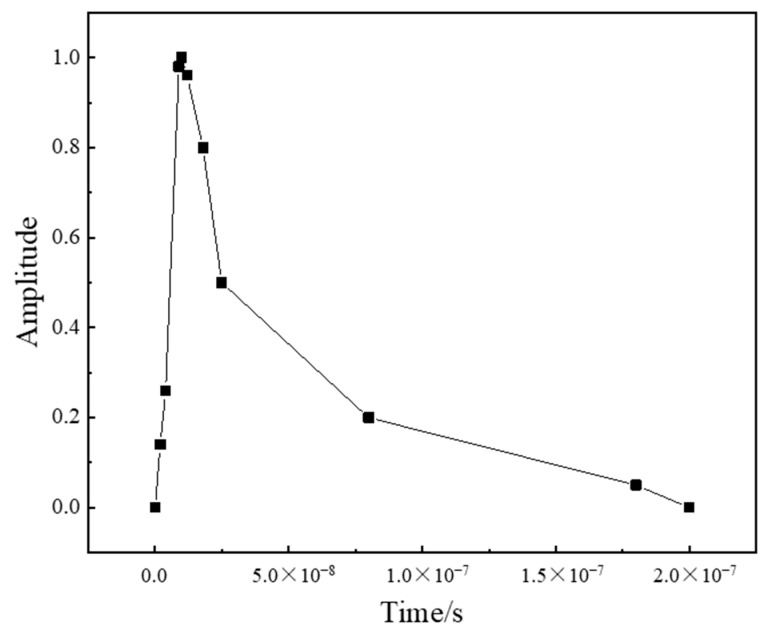
Shock wave pressure loading curve.

When constructing the laser shock wave pressure model, not only the spatial distribution of the shock wave needs to be considered, but also the temporal distribution. The simulation process in this paper uses a flat-top beam, and the expression for shock wave pressure is shown in Equation (3).

(3) $$P=P_{\max}\cdot P(x,y)\cdot P(t)$$

where *P* is the shock wave pressure; *P*_max_ is the peak pressure of the shock wave (the expression is shown in Equation (4) [19]); *P*(*x*, *y*) is the spatial distribution of the shock wave pressure (the specific expression is shown in Equation (8)), Figure 3 is the spatial distribution of the laser shock wave pressure; *P*(*t*) is the temporal distribution of the shock wave pressure, and the temporal amplitude curve is shown in Figure 4 [20].

(4) $$P_{\max}=0.01{\left(\frac{\xi }{2\xi +3}\right)}^{0.5}Z^{0.5}I^{0.5}$$

(5) $$I=\frac{4Q_{\;}}{\pi d^{2}\tau }$$

(6) $$\frac{2}{Z}=\frac{1}{Z_{T}}+\frac{1}{Z_{W}}$$

where *ξ* takes the value of 0.18, *I* is the laser power density, *Q* is the energy of the laser, *d* is the spot size, *τ* is the pulse width width, *Z* is the folded acoustic impedance of the specimen and the restraining layer, *Z*_W_ is the acoustic impedance of the water, g/(cm^2^s), *Z*_W_ = 0.165 × 10^6^ g/(cm^2^s), and *Z*_T_ is the acoustic impedance of the target material, g/(cm^2^s). The calculation is shown in Equation (7).

(7) $$Z_{T}=\sqrt{\rho E}$$

where *ρ* is the density of the metal material, kg/m^3^; *E* is the modulus of elasticity of the metal material, Pa.

(8) $$P(x,y)=\exp\left[-2\times {\left(\frac{\sqrt{x^{2}+y^{2}}}{d}\right)}^{10}\right]$$

where (*x*, *y*) is the spatial location point of the light field intensity; *d* is the spot size.

#### 2.2.2. Material Constitutive Model

During the laser shock process, the action time of the shock wave is very short, and the strain rate is also very high. The J-C (Johnson–Cook) model has a simple structural form and good consistency with experimental results [21,22,23,24]. The formula for the J-C model is shown in Equation (9) [25,26].

(9) $$\sigma_{y}=(A+B{\left(\varepsilon_{p}\right)}^{n})[1+CIn(\frac{{\dot{\varepsilon }}_{p}}{{\dot{\varepsilon }}_{0}})][1-{\left(T-T_{0}\right)}^{m}/{\left(T_{m}-T_{0}\right)}^{m}]$$

where *σ_y_* is the flow stress, MPa; *A* denotes the reference strain rate and the yield stress at the initial temperature, MPa; *B* is the strain hardening modulus, MPa; *n* is the hardening index; *C* is the strain rate strengthening parameter; *m* denotes the thermal softening index; *T*_m_ is the melting temperature of the material, °C; ${\dot{\varepsilon }}_{p}$ is the plastic strain rate, ${\dot{\varepsilon }}_{0}$ is the reference strain rate, and *T*_0_ is the room temperature, °C.

Since the metal surface is separated by a constrained protective layer, the temperature effect is usually ignored, and the J-C equation is simplified to Equation (10), and the material parameters of aluminum alloy 6082-T6 are shown in Table 1 [27].

(10) $$\sigma_{y}=(A+B{\left(\varepsilon_{p}\right)}^{n})[1+CIn(\frac{{\dot{\varepsilon }}_{p}}{{\dot{\varepsilon }}_{0}})]$$

### 2.3. Design of Plugin Program for Aluminum Alloy 6082-T6

#### 2.3.1. Dynamic Display Model’s Secondary Development

**Figure 5:**
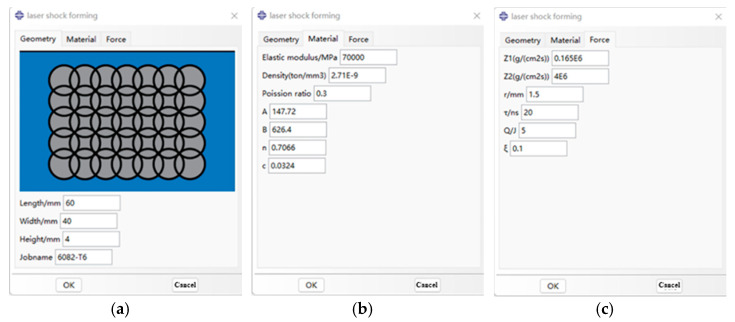
Aluminum alloy 6082-T6 explicit dynamic model plugin interface. (**a**) Dimensional parameters; (**b**) material parameters; (**c**) impact load parameters.

The graphical interface of the dynamic explicit plugin program is shown in Figure 5. The interface parameter options are constructed by the RSG (Really Simple and Graphical User Interface) dialog builder, and then the schematic diagram is imported into the plugin interface. The plugin is divided into three interfaces, namely, model size parameters, material property parameters, and laser impact load parameters. The dynamic explicit plugin program provides an intuitive and easy-to-use interface, allowing users to conveniently set model size parameters, material property parameters, and laser impact load parameters for relevant simulations and analyses.

#### 2.3.2. Implicit Static Layered Shell Model’s Secondary Development

**Figure 6:**
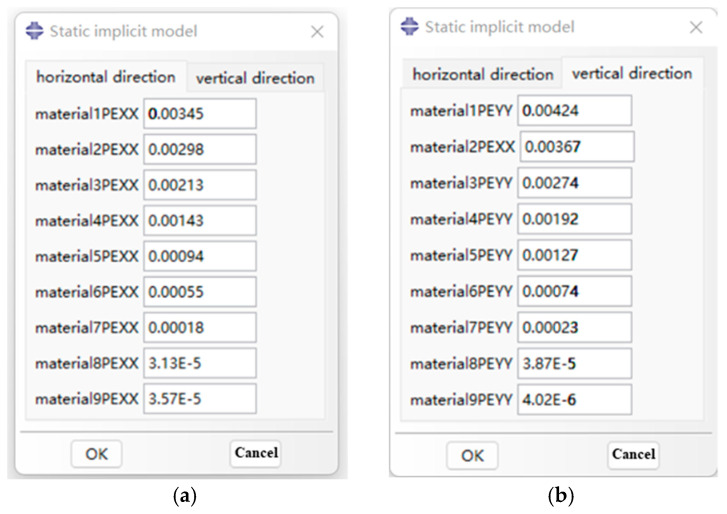
Interface of aluminum alloy 6082-T6 implicit static model plugin. (**a**) Transverse intrinsic strain; (**b**) longitudinal intrinsic strain.

The graphical interface of the inherent strain extraction plugin program is shown in Figure 6. The interface parameter options are constructed by the RSG dialog builder. The plugin interface is mainly divided into two parts: horizontal thermal expansion coefficient and vertical thermal expansion coefficient.

### 2.4. Model Geometry and Meshing

**Figure 7:**
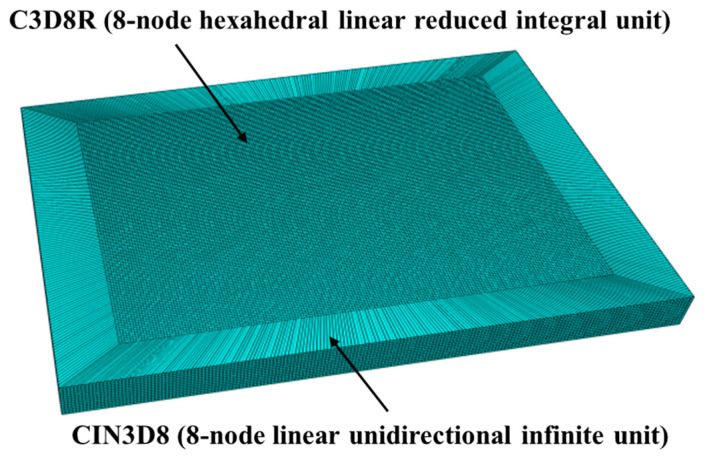
Finite element model after meshing.

**Figure 8:**
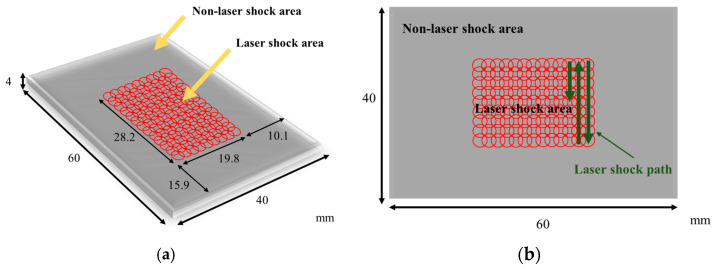
Laser shock forming simulation model. (**a**) Laser shock target size; (**b**) laser shock path and overlap method.

Python parameters can be used to create model geometry, followed by the selection of C3D8R (8-node hexahedral linear reduced integral unit) as the element type for the laser impact area, with a size of 0.18 mm and a depth of 0.09 mm. To avoid stress wave reflection, infinite element CIN3D8 (8-node linear unidirectional infinite unit) can be selected as the stress wave reflection boundary on the boundary of the laser impact area. The number of grid divisions is 598,000, and the finite element model after grid division is shown in Figure 7. The size of the laser shock model as well as the shock path are shown in Figure 8.

### 2.5. Methodology for Experimental Validation of Finite Element Models

**Figure 9:**
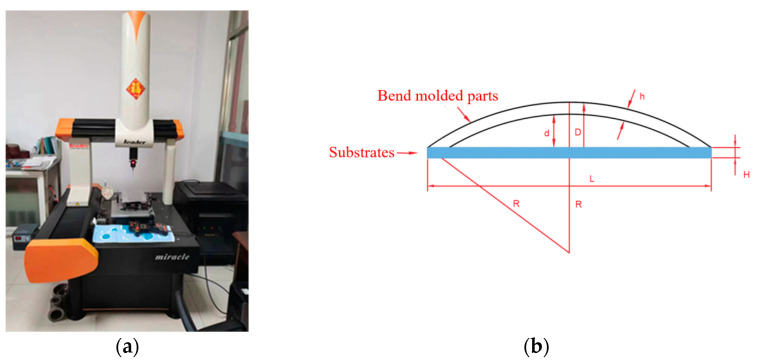
Measuring instruments and measuring methods. (**a**) Coordinate measuring machine; (**b**) arc bow height schematic.

After the plate is laser impacted, the plate will be bent to a certain extent. In this thesis, the coordinates of the deformed flat plate are measured using a coordinate measuring machine, and the calculated bow arc height is compared with the numerical values obtained from the simulation model in order to calibrate the bending and forming volume of the plate and to verify the accuracy of the finite element model. The three-coordinate measuring machine is shown in Figure 9a, and the measurement principle is shown in Figure 9b. The aluminum alloy’s plate thickness is h, the thickness of the substrate is H, the length of the substrate is L, the radius of curvature of the plate after bending is R, the length of the substrate L is 300 mm, the height of the arc bow is d, and the maximum distance between the bending plate and substrate is D.

## 3. Results and Analysis

### 3.1. Finite Element Model Verification

**Figure 10:**
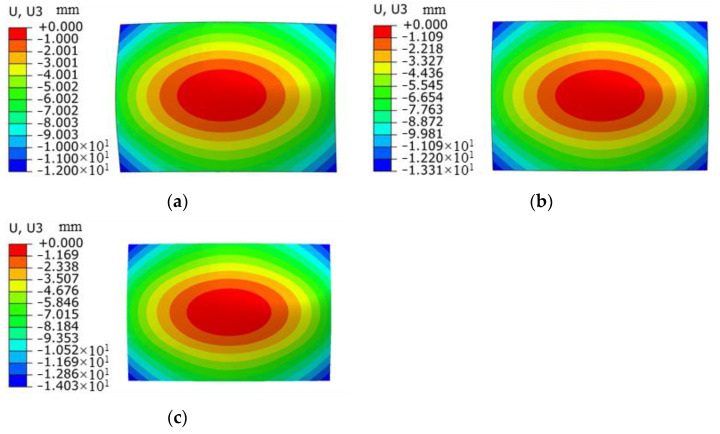
Simulation results of bending and forming volumes of aluminum alloy 6082-T6 under different laser energies. (**a**) Energy 5 J; (**b**) energy 6 J; (**c**) energy 7 J.

The laser pulse width is 20 ns, the spot size is 3 mm, the spot overlap rate is 30%, the plate thickness is 4 mm, and the Nd:YAG laser shock device is used to perform laser shock forming on the aluminum alloy 6082-T6 plate with energies of 5 J, 6 J, and 7 J, respectively. Figure 10 shows the simulation results of the bending and forming volumes of aluminum alloy 6082-T6 under different laser energies, and the arc bow heights are 6.6 mm, 7.2 mm, and 7.6 mm under laser energies of 5 J, 6 J, and 7 J, respectively.

**Figure 11:**
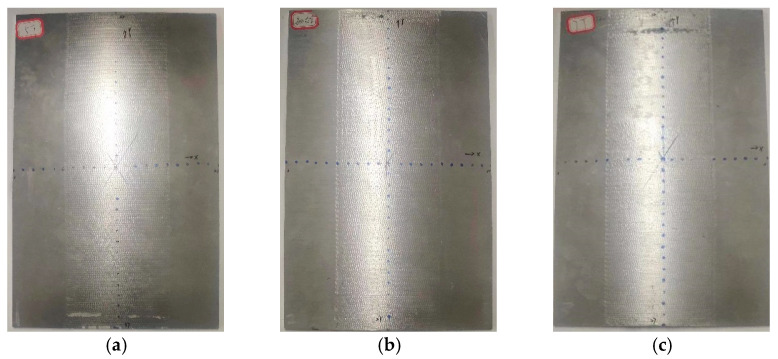
Deformation diagrams of 6082-T6 aluminum alloy flat plates at different laser energy levels. (**a**) Energy 5 J; (**b**) energy 6 J; (**c**) energy 7 J.

**Figure 12:**
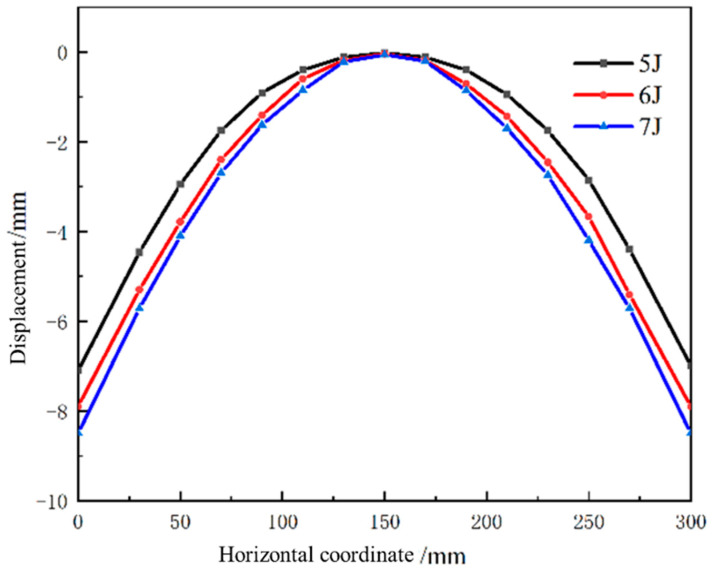
Forming amounts of 6082-T6 aluminum alloy flat plates at different laser energy levels.

Figure 11 shows the deformation of the experimental part of aluminum alloy 6082-T6 at different laser energies. Figure 12 shows the forming amount of aluminum alloy 6082-T6 flat plates at different laser energies. From the figure, it can be seen that the arc bow heights of aluminum alloy 6082-T6 experiments are 7.1 mm, 7.5 mm and 7.9 mm at laser energies of 5 J, 6 J and 7 J, respectively.

**Figure 13:**
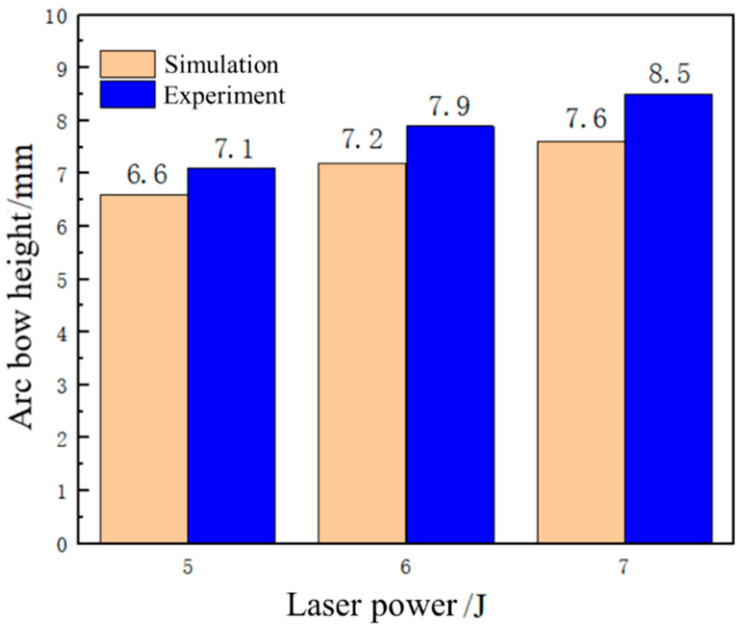
Comparison of 6082-T6 aluminum alloy arch height simulation and experiment at different laser energy levels.

Figure 13 is a comparison between the simulation and experimental results of the arc height of aluminum alloy 6082-T6 at different laser energies. The errors between the simulation results and the experimental results are approximately 7.5%, 9.7%, and 11.8%, indicating that the accuracy of the simulation using the plugin is high. Meanwhile, the experimental results and simulation data show that the laser energy has a significant effect on the molding volume of the plate, and with the increase in laser energy, the molding volume of the plate increases. This is because as the laser power density increases, the generated shock load also increases, resulting in a more intense shock wave and greater plastic deformation of the plate.

### 3.2. Effect of Plate Thickness on Forming Quantity of the Plate

**Figure 14:**
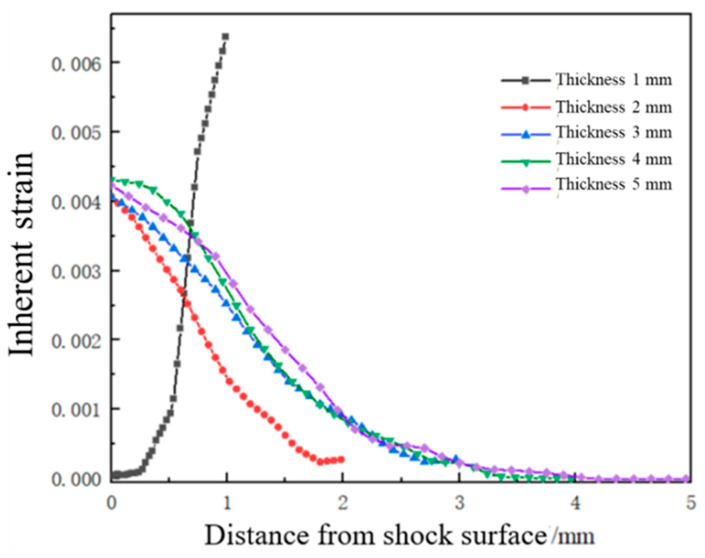
Plastic strain distribution along the depth direction of the target material under different plate thicknesses.

**Figure 15:**
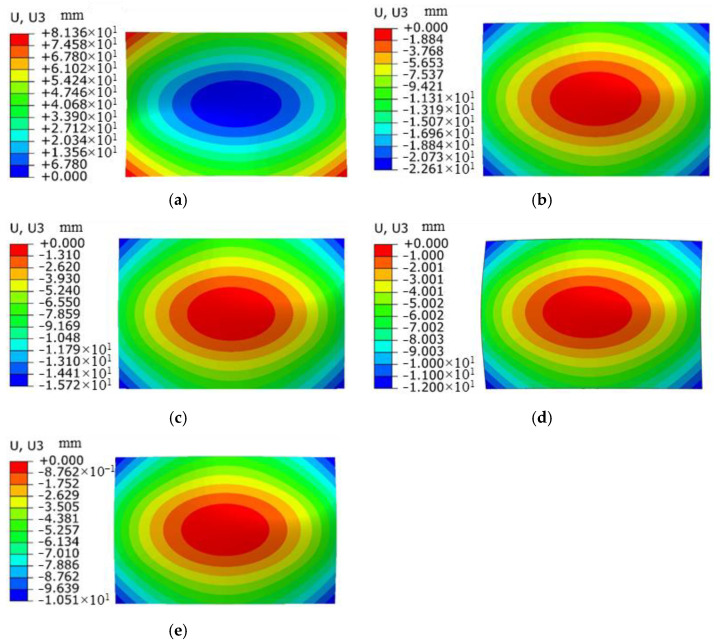
Simulation results of forming amount for aluminum alloy 6082-T6 flat plates with different plate thicknesses. (**a**) Thickness 1 mm; (**b**) thickness 2 mm; (**c**) thickness 3 mm; (**d**) thickness 4 mm; (**e**) thickness 5 mm.

**Figure 16:**
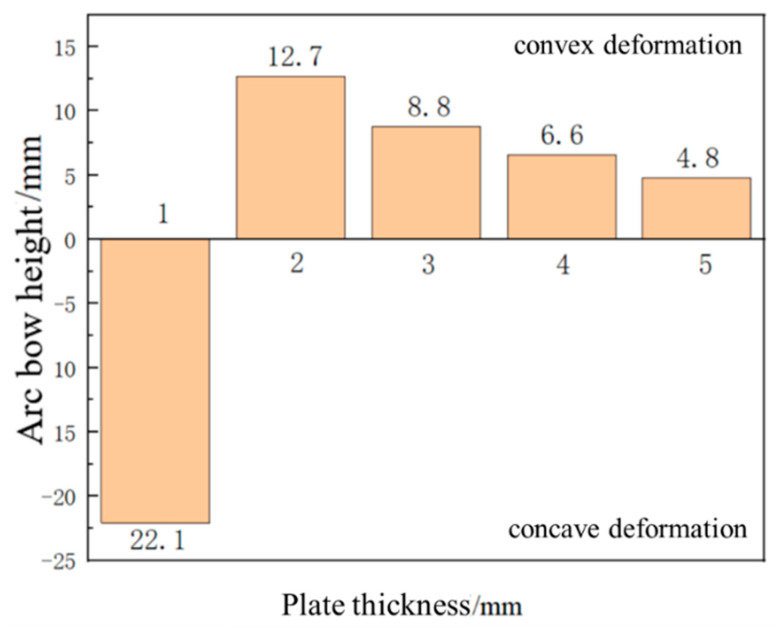
Arching height of aluminum alloy 6082-T6 flat plates with different plate thicknesses.

**Figure 17:**
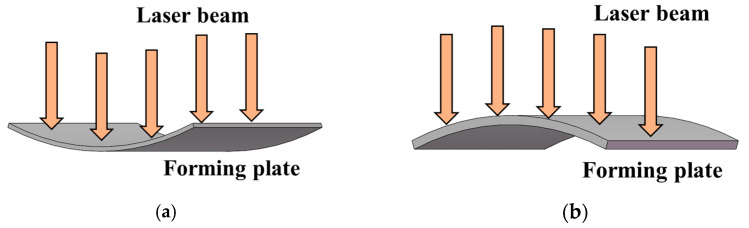
Deformation mode of forming plates. (**a**) Concave deformation; (**b**) convex deformation.

With a laser energy of 5 J, a spot size of 3 mm, a spot overlap rate of 30%, and a laser pulse width of 20 ns, under the condition that the other parameters remain unchanged, laser shock forming processing is performed on plates with thicknesses of 1 mm, 2 mm, 3 mm, 4 mm, and 5 mm, respectively. The distribution curve of plastic strain along the thickness direction is extracted, as shown in Figure 14. Figure 15 is a simulation diagram of the plate under different thicknesses, and Figure 16 is the arc height of the plate under different thicknesses. From the figures, it can be seen that when the plate thickness is 1 mm, the initial plastic strain is the smallest, then increases monotonically to the maximum value, and the plastic strain on the bottom surface of the plate is the largest, while the plastic strain on the laser impact surface is the smallest. The plate undergoes concave deformation in the opposite direction of the laser impact, and the arc height of the plate is 22.1 mm. This is because when the plate is thin, the laser-induced shock wave propagates along the thickness of the plate without significant attenuation. The pressure generated by the shock load far exceeds the yield strength of the material, and the aluminum alloy is unable to absorb all of the plastic strain energy resulting from the shock. The shock load produces downward inertia in the impact region, causing the plate to deform plastically in the thickness direction, resulting in the deformation of the plate from the backside of the impact, as shown in Figure 17a. When the plate thickness is 2 mm, 3 mm, 4 mm, and 5 mm, the initial plastic strain of the plate is the largest and gradually approaches 0. The plastic strain on the laser impact surface of the plate is larger, and the plastic strain on its impact back surface is smaller. The plate bends in the direction of the impact, forming a convex deformation, as shown in Figure 17b. Due to the rapid attenuation of the laser shock wave propagating in the thickness direction, a concave pit is formed on the impact surface of the plate. Restricted by the surrounding materials, the impact area forms residual stress, and a steep stress gradient forms inside the plate, gradually decreasing in amplitude, resulting in the formation of convex deformation. As the plate thickness increases, the arc height decreases, with values of 12.7 mm, 8.8 mm, 6.6 mm, and 4.8 mm, respectively. If the same bending deformation is achieved, plates with larger thicknesses require larger moments, so the arc height of the plate decreases gradually.

### 3.3. Effect of Spot Size on Forming Quantity of the Plate

**Figure 18:**
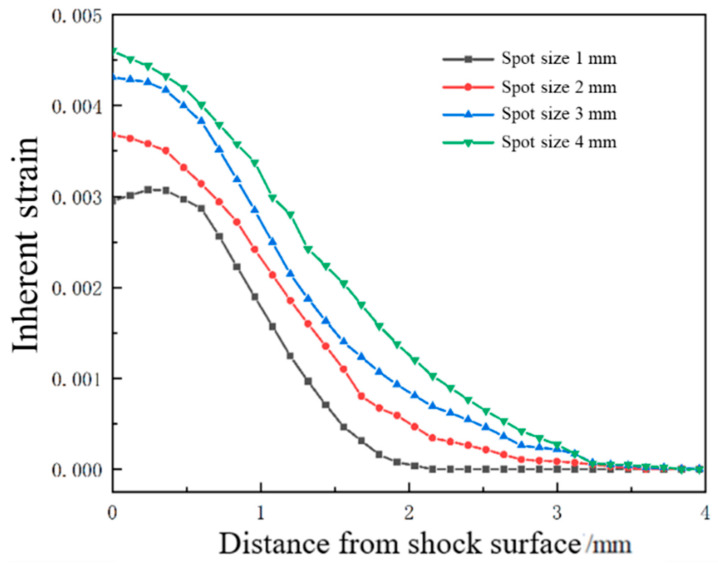
Plastic strain distribution along the depth direction of the target material under different beam sizes.

**Figure 19:**
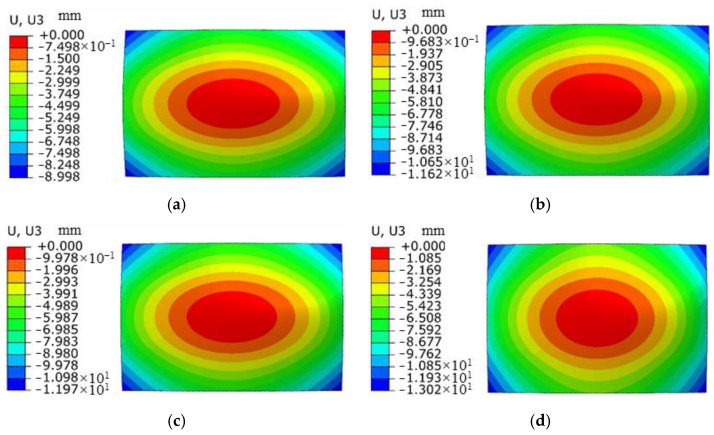
Simulation diagram of plate deformation under different spot sizes. (**a**) Spot 1 mm; (**b**) spot 2 mm; (**c**) spot 3 mm; (**d**) spot 4 mm.

**Figure 20:**
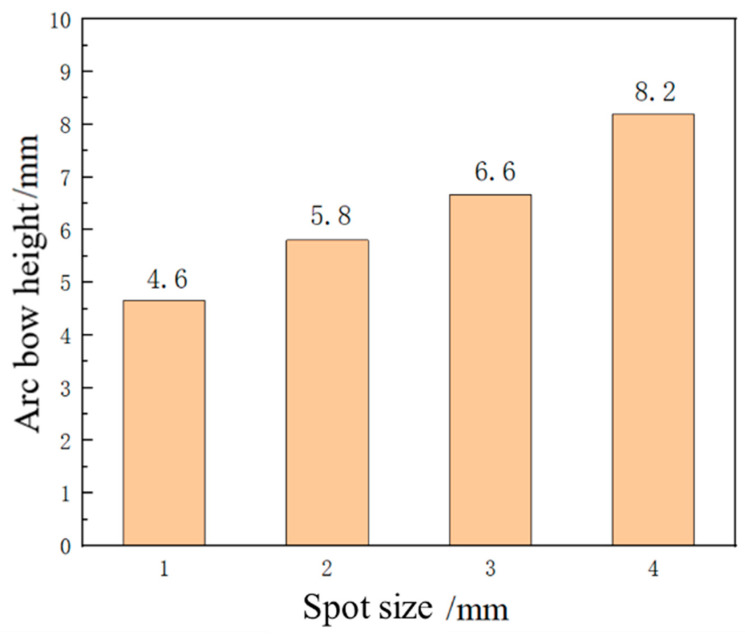
Arching height of aluminum alloy 6082-T6 flat plates under different laser spot sizes.

The laser energy is 5 J, the laser pulse width is 20 ns, and the thickness of the aluminum alloy 6082-T6 sheet is 4 mm. Under the condition that the other parameters remain unchanged, the aluminum alloy 6082-T6 sheet is subjected to impact forming using spot sizes of 1 mm, 2 mm, 3 mm, and 4 mm, respectively. The distribution curve of plastic strain in the thickness direction is shown in Figure 18. The trend of plastic strain curve changes similarly with the increase in spot size, and the plastic strain value increases correspondingly. Due to the maximum plastic strain on the impact surface and the minimum plastic strain on the bottom surface, the sheet bends in a convex shape. Figure 19 shows the simulation diagram of the sheet under different spot sizes. The arc height of the sheet under different spot sizes is 4.6 mm, 5.8 mm, 6.6 mm, and 8.2 mm, as shown in Figure 20. It can be seen from the figure that with the increase in spot size, the degree of sheet deformation also increases. This is because the shock wave formed by a large spot size propagates as a plane wave, while the shock wave formed by a small spot size propagates as a spherical wave. Plane waves attenuate slower during energy transmission, which can generate larger residual pressure stress and larger forming amounts on the sheet. In contrast, spherical waves attenuate faster during energy transmission, resulting in smaller residual pressure stress and smaller forming amounts on the sheet. In practical applications, the bending forming amount of the sheet can be adjusted by controlling the size of the spot.

### 3.4. Effect of Overlap Ratio on Forming Quantity of the Plate

**Figure 21:**
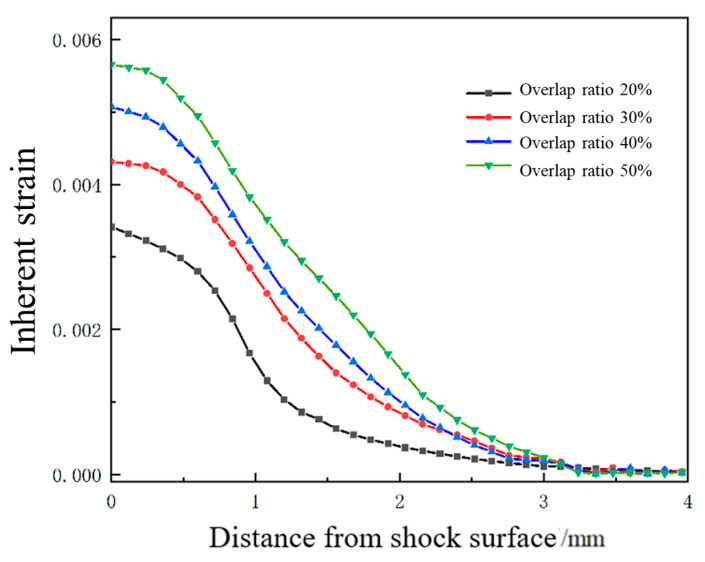
Plastic strain distribution along the depth direction of the target material under different overlap rates.

**Figure 22:**
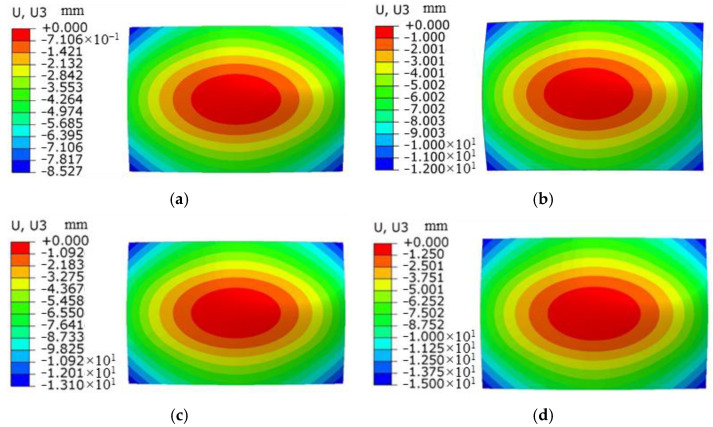
Simulation results of forming amount for aluminum alloy 6082-T6 flat plates at different lap ratios. (**a**) 20%; (**b**) 30%; (**c**) 40%; (**d**) 50%.

**Figure 23:**
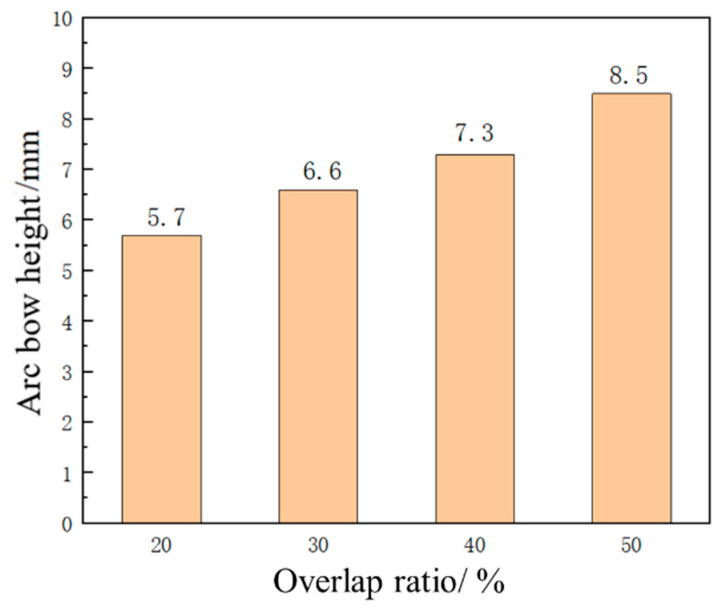
Arching height of aluminum alloy 6082-T6 flat plates at different lap ratios.

In the laser shock forming process, the overlap ratio has an important influence on plastic strain and forming amount of the sheet. With the laser pulse width of 20 ns, laser energy of 5 J, and spot size of 3 mm, the sheet with a thickness of 4 mm is impacted by a laser with overlap ratios of 20%, 30%, 40%, and 50%. The distribution curve of plastic strain along the thickness direction at the center position of the sheet is extracted as shown in Figure 21, and the change trend of plastic strain under different overlap ratios is analyzed. For different laser overlap ratios, the change trend of plastic strain is similar, and the decrease in strain is relatively slow within the depth range of 600 μm. It gradually decreases with the increase in depth. It is worth noting that with the increase in the overlap ratio, the plastic strain value also increases. Due to the maximum plastic strain on the impact surface and the minimum plastic strain on the bottom surface, the sheet exhibits convex deformation. Figure 22 shows the simulation diagram of the sheet under different overlap ratios. Figure 23 shows the comparison of arc height under different overlap ratios, which are 5.7 mm, 6.6 mm, 7.3 mm, and 8.5 mm, respectively. With the increase in overlap ratio, the arc height of the sheet gradually increases. The increase in overlap ratio results in an increase in the overlap at the center of the circle between two adjacent spots. With more spot overlap of neighboring laser pulses, more energy is accumulated on the surface of the material. This leads to a more intense energy density in the shock region and plastic strain energy accumulates in the overlap region, which causes greater deformation of the aluminum alloy.

## 4. Conclusions

Taking aluminum alloy 6082-T6 flat plates as the research object, based on the ABAQUS secondary development and the intrinsic strain theory, a parameterized plugin for the laser forming process was developed using Python. Simulations and research under different process parameters were conducted using this plugin. The specific conclusions are as follows:

(1) The plugin can quickly establish an explicit dynamic model and extract the distribution of intrinsic strain along the depth direction of the characteristic unit. Then, it is applied to an implicit static model, and the bending forming amount of the sheet after laser impact forming is predicted through elastic analysis. The simulation results have small errors.
(2) The laser energy has a large effect on the amount of plate formed, which increases as the laser energy increases.
(3) When the thickness of the sheet is 1 mm, the sheet undergoes concave deformation in the direction of the impact. With the increase in thickness, the bending forming amount of the sheet decreases, and the sheet exhibits convex deformation.
(4) Under the same laser power density, the forming amount of the sheet increases with the increase in spot size.
(5) With the increase in the overlap ratio, the arc height of the sheet gradually increases.

## Figures and Tables

**Table 1:** The material properties of alloy 6082-T6.

Material	*ρ* g/cm^3^	*E*/GPa	Poisson’s Ratio	*A*/MPa	*B*/MPa	*n*	*C*
6082-T6	2.7	70	0.3	274.65	169.98	0.2806	0.02

## Data Availability

The data are available within the article.
